# Experts’ preferences for sarcopenia outcomes: a discrete-choice experiment from a working group of the European Society for Clinical and Economic Aspects of Osteoporosis, Osteoarthritis and Musculoskeletal Diseases (ESCEO) in collaboration with the European Union of Geriatric Medicine Society (EUGMS)

**DOI:** 10.1007/s40520-021-01794-2

**Published:** 2021-03-05

**Authors:** Charlotte Beaudart, Jürgen M. Bauer, Francesco Landi, Olivier Bruyère, Jean-Yves Reginster, Mickael Hiligsmann, ESCEO Working Group on Sarcopenia, Sarcopenia Interest Group of the EUGMS

**Affiliations:** 1 WHO Collaborating Center for Public Health Aspects of Musculo-Skeletal Health and Ageing, Division of Public Health, Epidemiology and Health Economics, University of Liège, Liège, Belgium; 2 Department of Health Services Research, CAPHRI Care and Public Health Research Institute, Maastricht University, Maastricht, the Netherlands; 3 Center for Geriatric Medicine and Network Aging Research (NAR), Heidelberg University, Heidelberg, Germany; 4 Department of Geriatrics, Neurosciences and Orthopedics, Catholic University of the Sacred Heart Rome, Milano, Italy

**Keywords:** Discrete-choice experiment, Sarcopenia, Outcomes

## Abstract

**Background and aims:**

To assess experts’ preference for sarcopenia outcomes.

**Methods:**

A discrete-choice experiment was conducted among 37 experts (medical doctors and researchers) from different countries around the world. In the survey, they were repetitively asked to choose which one of two hypothetical patients suffering from sarcopenia deserves the most a treatment. The two hypothetical patients differed in five pre-selected sarcopenia outcomes: quality of life, mobility, domestic activities, fatigue and falls. A mixed logit panel model was used to estimate the relative importance of each attribute.

**Results:**

All sarcopenia outcomes were shown to be significant, and thus, important for experts. Overall, the most important sarcopenia outcome was falls (27%) followed by domestic activities and mobility (24%), quality of life (15%) and fatigue (10%).

**Discussion and conclusion:**

Compared to patient’s preferences, experts considered falls as a more important outcome of sarcopenia, while the outcomes fatigue and difficulties in domestic activities were considered as less important.

## Introduction

Sarcopenia, defined by the European Working Group on Sarcopenia in Older People (EWGSOP2) [1] as a loss of muscle strength combined with a loss of muscle mass, is now recognized to engender multiple health consequences, both at the individual as well as the societal level [2]. Within a patient-centered care approach, Hiligsmann et al. conducted, in 2019, a discrete-choice experiment (DCE) [3] among 216 sarcopenic patients from 6 European countries to identify the most important outcomes for patients. Results highlighted decreased mobility and difficulties with domestic activities as the two most important sarcopenia-related outcomes for patients.

Besides the patients’ point of view, assessing the preferences of experts (including healthcare professionals) for sarcopenia outcomes would be interesting, especially as discordance in preferences between patients and professionals for healthcare observations have been regularly observed [4]. Particularly for outcomes preferences, it has been shown, for example, that healthcare providers and patients could be concordant on the importance of outcomes such as quality of life but discordant on mortality as a relevant outcome, the latter being systematically rated as much more important by healthcare providers than by patients [4, 5]. DCE studies provide an excellent opportunity to determine whether there is concordance of preferences between patients and health care providers. The preferences of healthcare professionals for specific outcomes may exert a relevant impact on the management of health resources, e.g., sarcopenic patient care.

This study aimed, therefore, to assess experts’ preference for sarcopenia outcomes through a DCE, and to compare experts’ preferences to patients’ preferences, which were identified by the previous study.

## Methods

**Fig. 1:**
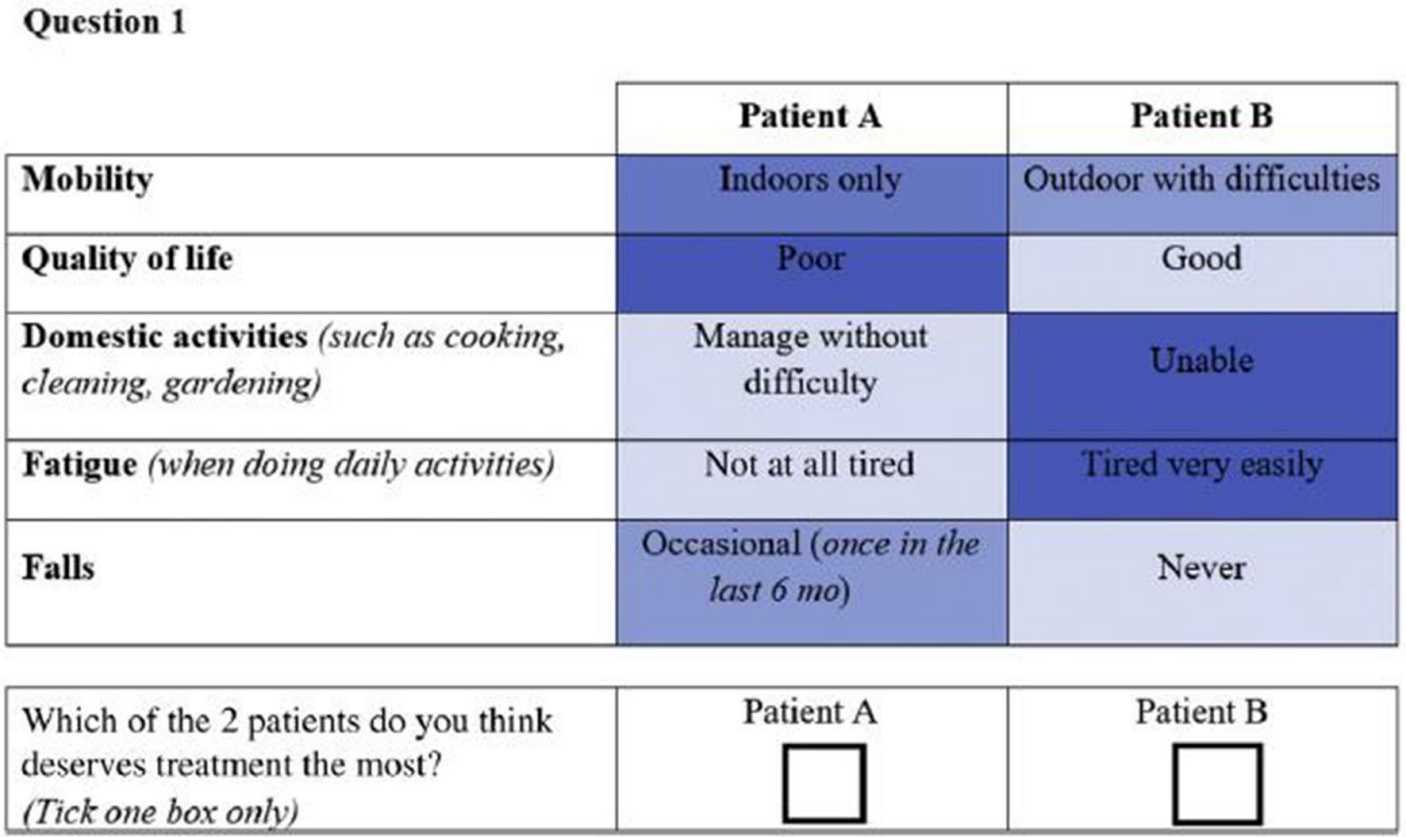
Example choice set of the DCE questionnaire

In the present study, we used the same DCE as was used to elicit patients’ preferences for sarcopenia outcomes [3]. In the DCE survey, participants were repetitively asked to choose which one of two hypothetical patients (Patient A and Patient B) suffering from sarcopenia deserves the most a treatment. The two hypothetical patients presented different levels of risk for five sarcopenia outcomes (i.e., the “attributes”): quality of life, mobility, domestic activities, fatigue and falls. More information regarding the full methodology of attributes and level selections for the sarcopenia outcomes presented in the choice sets can be found in previous publications [3, 6]. Briefly, a literature review, an expert consultation, a focus group with participants suffering from sarcopenia and an expert meeting have been conducted to identify both the attributes and the levels relevant for the DCE. Attributes and levels included in the DCE are displayed in Table 1. Two versions of the questionnaire were randomly proposed to participants. A total of 24 choice tasks were designed and blocked into these 2 versions containing 12 choice tasks each. An example of choice set is presented in Fig. 1. A dominance test, i.e., a choice set with one hypothetical patient who has clearly better outcomes than the other, was added to the questionnaire to assess the reliability of respondents’ choices. Patients who failed the dominance test were excluded from the analyses. The full questionnaire is available on request to the corresponding author.

**Table 1:** Attributes and levels included in the DCE

Attributes	Levels
Patient’s mobility	Outdoor mobility without difficulties
	Outdoor mobility with difficulties
	Indoor mobility only
	Chairbound or bedbound
Patient’s quality of life	Good
	Fair
	Poor
Patient’s management of domestic activities	Manages without difficulties
	Manages with difficulty
	Unable
Patient’s level of fatigue	Not at all tired
	Moderately tired
	Tired very easily
Frequency of falls	Never
	Occasional (once in the last 6 mo)
	Frequent (2 or more times in the last 6 mo)

Sarcopenia experts involved in the present DCE were recruited, in 2019, among experts from the Special Interest Group in Sarcopenia from the European Geriatric Medicine Society (EUGMS). The SIG comprised 84 international European and non-European healthcare professionals, e.g., medical doctors specialized in geriatrics, nutrition or physical rehabilitation, as well as researchers working in the field of sarcopenia. Participants were invited by email and the DCE questionnaire was developed in an online self-administered format using Qualtrics (Qualtrics, Provo, UT, USA). Data on participants’ demographics and socioeconomic characteristics were collected, e.g., sex, age, workplace, specialization and year of experience.

Data analysis was carried out using Nlogit software, version 5.0. In line with the patients DCE, a mixed logit panel model was used to estimate the relative importance of each DCE attribute for the experts. Standard deviation significantly different from zero was interpreted as evidence of significant preference heterogeneity for the attributes and levels in the sample. The relative importance of each outcome was calculated by dividing the attribute-specific level range by the sum of all attribute level ranges.

## Results

Among 84 members of the SIG sarcopenia group of the EUGMS, 37 (44.0%) completed the questionnaire, but 4 participants failed the dominance test and were, therefore, excluded. The remaining 33 respondents were gender balanced with 51.5% being female and 48.5% being male. The majority of the respondents were medical doctors (66.7%), while some researchers (24.2%) as well as Ph.D. students in the field of sarcopenia (9.1%) also completed the survey. The median professional experience of experts was 9 years (3–15 years). Experts were recruited from various countries in Europe and across the world: Belgium (*n* = 6), France (*n* = 3), Germany (*n* = 1), Italy (*n* = 1), Netherlands (*n* = 2), Poland (*n* = 1), Spain (*n* = 4), Sweden (*n* = 1), Switzerland (*n* = 3), Turkey (*n* = 5), Czech Republic (*n* = 1), UK (*n* = 1), Australia (*n* = 1), Saudi Arabia (*n* = 1) and USA (*n* = 2).

Results of the mixed logit model are reported in Table 2. All five pre-selected sarcopenia outcomes were statistically significant, and thus, important for experts. The signs of coefficient were as excepted, and the standard deviations were significant for patients’ mobility, patients’ management of domestic activities and frequency of falls, indicating variability in the respondents’ preferences. Overall, the most important sarcopenia outcome for experts was falls (27%) followed by patient’s management of domestic activities and patient’s mobility (24%), patient’s quality of life (15%) and fatigue (10%).

**Table 2:** Results from the Panel Mixed Logit Model

Attributes and levels	Estimate (95% CI)	Standard deviation	Relative importance (%)
Patient’s mobility			24%
Outdoor mobility without difficulties	− 1.59 (− 2.53;− 0.66)**	1.11*	
Outdoor mobility with difficulties	0.81 (0.05;1.58)*	1.27**	
Indoor mobility only	0.66 (− 0.03;1.36)	0.51	
Chairbound/bedbound	0.1179 (− 0.93;1.17)		
Patient’s quality of life			15%
Good	− 0.82 (− 1.37;− 0.27)**	0.39	
Fair	0.12 (− 0.25;0.49)		
Poor	0.70 (0.18;1.2)**	0.43	
Patient’s management of domestic activities			24%
Manages without difficulties	− 1.61 (− 2.52;− 0.71)**	0.57	
Manages with difficulty	0.79 (0.16;1.41)*		
Unable	0.82 (0.19;1.46)*	0.84**	
Patient’s level of fatigue			10%
Not at all tired	− 0.52 (− 1.01;-0.02)*	0.19	
Moderately tired	− 0.03 (− 0.36;0.29)		
Tired very easily	0.54 (0.01–1.07)*	0.01	
Frequency of falls			27%
Never	− 1.40 (− 2.25;− 0.56)**	0.67**	
Occasional (once in the last 6 months)	0.02 (− 0.43;0.47)		
Frequent (2 or more times in the last 6 months)	1.38 (0.58–2.18)**	0.77**	

^*^P < 0.05

^**^P < 0.01

## Discussion

This DCE study highlights that experts valued falls as the most relevant outcome. Compared to a similar study conducted with patients suffering from sarcopenia, some differences in the relative importance of sarcopenia outcomes were observed. Experts considered falls as more important that patients did (27% versus 18%), while fatigue (10% versus 17%) and mobility (24% versus 30%) were less important for experts. Both experts and patients seem, however, concordant about the level of importance of quality of life and the ability of managing domestic activities as outcomes of sarcopenia.

Different reasons could explain the differences between experts and patients on outcome ranking. Falls, for example, could have been considered more important by experts as they are well aware of the potential burden of falls from the perspective of the individual as well as from a public health point of view. Falls can be dramatic and have been shown to increase the risk of injuries, hospitalization, institutionalization, healthcare costs, and death [7–9]. They are also related to a reduction of quality of life, which could partially explain their higher ranking. Indeed, quality of life could be considered as a consequence of other outcomes [10]. Falls may be regarded as an objective clinical outcome that could be measured in future studies.

Patients, on the other side, seem to be more concerned about outcomes that directly impact their daily life, such as fatigue or mobility limitations, which can restrict themselves with regards to their independence, social interactions and which can also directly or indirectly impact other outcomes such as the ability to perform domestic activities and quality of life [11]. Fatigue was a more important outcome for patients, but showed the lowest ranking among experts. Fatigue related to sarcopenia may be considered by clinicians as a natural consequence of sarcopenia without a major impact on individual health or society. When the five outcomes were generated from the literature review, the patients’ perspective and the experts’ perspective, this outcome was not identified at all by the experts’ panel, probably because of the highly subjective nature of this outcome that is only felt by the patients themselves.

To our knowledge, this study provides the first assessment of experts’ preferences on sarcopenia outcomes and it revealed some differences between experts and patients. Results of such DCE identifying the most relevant outcomes of sarcopenia could be useful for the choice of primary and secondary endpoints of future interventional studies [12]. As experts and patients assigned different priorities to outcomes of sarcopenia, it is highly important to take both perspectives into account.

However, some potential limitations of this study exist. First, only a restricted sample size of experts was included, which could limit the generalizability of our results. Even if there is no specific power size recommended for conducting DCE, de Bekker-Grob [13] published a review in 2015 that showed among a sample of 69 DCEs performed in Healthcare, that a mean of 100–300 respondents is often targeted for DCE, which is noticeably higher than our sample size. Despite a moderate sample size, we nevertheless tried to be as much representative as possible by including heterogeneous profiles of experts from different European and non-European countries. Second, DCEs are evaluating hypothetical options. It is, therefore, impossible to guarantee that responders will apply their choices in real life.

## Conclusion

Sarcopenia experts and patients suffering from sarcopenia assigned different priorities to outcomes associated with this condition. This discordance highlights the importance of considering both perspectives for the choice of primary and secondary outcomes in future intervention trials.

## Data Availability

Under request to the corresponding author.

## Code availability

Under request to the corresponding author.
